# Clinical implications and risk factors for QRS prolongation over time in heart failure patients

**DOI:** 10.1007/s00392-022-02122-y

**Published:** 2022-11-15

**Authors:** Martin Berger, Nina Kumowski, Sam Straw, Marlo Verket, Nikolaus Marx, Klaus K. Witte, Katharina Schütt

**Affiliations:** 1 Department of Internal Medicine I, University Hospital Aachen, RWTH Aachen University, Aachen, Germany; 2 Leeds Institute of Cardiovascular and Metabolic Medicine, University of Leeds, Leeds, UK

**Keywords:** QRS width progression, Heart failure, NTproBNP, ECG

## Abstract

**Background:**

QRS prolongation is an established prognostic marker in heart failure (HF). In contrast, the role of QRS width progression over time has been incompletely explored. The current study investigates the role of QRS width progression over time on clinical status and identifies underlying predictors.

**Methods:**

Datasets of ≥ 2 consecutive visits from 100 attendees to our HF clinic between April and August 2021 were analysed for changes in QRS complex duration.

**Results:**

In total 240 datasets were stratified into tertiles based on change in QRS duration (mm/month) (1st tertile: − 1.65 [1.50] ‘regression’; 2nd tertile 0.03 [0.19] ‘stable’, 3rd tertile 3.57 [10.11] ‘progression’). The incidence of the combined endpoint HF hospitalisation and worsening of symptomatic heart failure was significantly higher in the group with QRS width progression (3rd tertile) compared with the stable group (2nd tertile; log-rank test: *p* = 0.013). These patients were characterised by higher plasma NT-pro-BNP levels (*p* = 0.008) and higher heart rate (*p* = 0.007). A spline-based prediction model identified patients at risk of QRS width progression when NT-pro-BNP and heartrate were > 837 pg/ml and > 83/bpm, respectively. These markers were independent of guideline-directed medical HF therapy. Patients beyond both thresholds had a 14-fold increased risk of QRS width progression compared to those with neither or either alone (HR: 14.2 [95% 6.9 – 53.6]; *p* < 0.0001, *p* for interaction = 0.016).

**Conclusions:**

This pilot study demonstrates that QRS width progression is associated with clinical deterioration of HF. NTproBNP plasma levels and heart rate indicate patients at risk QRS width progression, independently of HF therapy.

**Graphic abstract:**

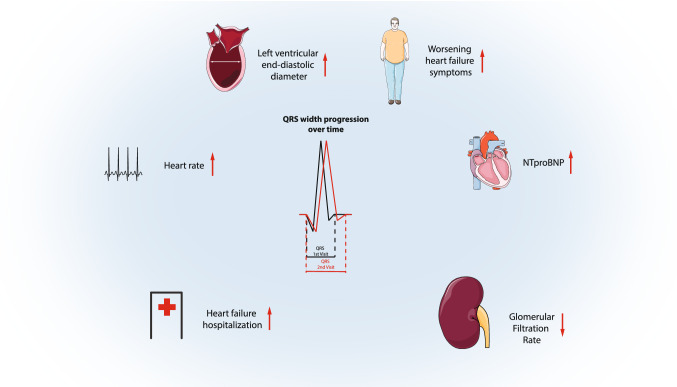

**Supplementary Information:**

The online version contains supplementary material available at 10.1007/s00392-022-02122-y.

## Introduction

Chronic heart failure is a growing public health problem, affecting 1–2% of the general adult population in developed countries [1]. Although the age-adjusted incidence is falling, the background of an aging population means the overall incidence is increasing [2]. QRS complex prolongation, particularly left bundle branch block > 120 ms, is an established prognostic marker in patients with heart failure due to reduced ejection fraction (HFrEF), and is independently associated with worsening symptoms, progressive remodelling less amenable to medical therapy [3], hospitalisation and all-cause mortality [4, 5]. For these patients, cardiac resynchronisation therapy (CRT) in combination with medical therapy can halt clinical progression and induce reverse remodelling, thereby improving symptoms and prognosis [6]. In patients with severe left ventricular (LV) systolic dysfunction (LVSD) but without QRS prolongation, an implantable cardioverter defibrillator (ICD) can provide protection against sudden arrhythmic death. A substantial proportion of patients with HFrEF (perhaps up to 30% [7]) who have narrow QRS develop progression of QRS width despite medical therapy [8]. This often occurs within the lifetime of the original device; more than one quarter of all CRT procedures are device upgrades [8], and almost 60% of all secondary procedures were upgrades to CRT [9], most commonly from single or dual chamber ICDs. However, predictors of progression and how these are associated with changes in clinical status have been incompletely explored. Given that device upgrade from ICD to CRT-D is the procedure most likely to be associated major complications [9] identification of those at heightened risk of QRS width progression, and applying predicted eligibility for CRT-D therapy to the first device prescription could have important benefits for the timeliness, safety and cost-effectiveness of the patients’ lifetime use of device therapy by avoiding upgrade procedures for some. Therefore, the present study aimed to provide pilot data that could help identify features associated with QRS width progression and its relationship with changing clinical status.

## Methods

The present retrospective analysis relates to 100 consecutive attendees to our hospital-based HF clinic between April and August 2021. Each patient with a least one past attendance including a resting electrocardiogram (ECG) was included. A maximum of 4 consecutive, past visits were assessed per patient. The mean time between visits was 227 (427) days. Patients with a cardiac implantable electronic device (CIED), or cardiac contractility modulation (CCM) devices and ventricular pacing ventricular pacing ≥ 0.1% were excluded. In addition to an ECG, standard care for each visit includes an assessment of symptoms, transthoracic echocardiography and blood tests including N-terminal B-type natriuretic peptide (NT-pro-BNP). All data pertaining to the present study were retrieved from the patient-management server of the University Hospital of Aachen and stored on a secure, bespoke database for analysis. Due to the anonymous retrospective analysis of routinely collected clinical data, informed consent was not required.

## Electrocardiography

All ECGs were performed with a Cardiovit MS-2010 (Schiller, Germany) at 50 mm/sec speed with an amplitude setting of 10 mm/mV by experienced technical personnel in the resting supine position and stored electronically on the patient management system. For the present study, these images were recalled and manually interpreted by two experienced physicians (MB or NK) to record heart rate, PR interval, QRS duration and morphology. Cardiac cycles immediately following atrial or ventricular ectopics were avoided. QRS duration was assessed in Lead V2 and V4 and an average was calculated.

## Echocardiography

Echocardiographic examinations were performed according to recommended standards of the American Society of the Echocardiography and the European Association of Cardiovascular Imaging [10] using a GE Vivid E95 system (GE Vingmed Ultrasound AS, Horten, Norway). Interventricular septum diameter (IVSd), LV end diastolic (LVEDD) and end systolic (LVESD) diameters were measured in the parasternal long-axis views. LV ejection fraction (LVEF) was derived from volume measurements using the biplane Simpson method and manual tracing of the digital images. Right ventricular systolic pressure (RVSP) was derived, where possible, from measurements of tricuspid regurgitation in the four-chamber view.

## Laboratory tests

Whole blood samples, including serum, EDTA plasma and citrated plasma, were routinely obtained at each visit and analysed according to the laboratory standards of University Hospital Aachen.

## Clinical outcomes

The primary outcome was prespecified as a combination of (i) hospitalisation for decompensated heart failure and (ii) deteriorating symptoms defined as an increase of at least one grade of the New York Heart Association (NYHA) classification. On each visit, NYHA class was assessed by a trained heart failure clinician based on structured assessment of dyspnoea, exercise capacity and physical activity.

## Statistical strategy

The primary aim of this pilot study was to identify factors that may influence QRS progression over time. Therefore, to increase the power and precision of our prediction models, datasets were generated per patient based on data from two consecutive visits (i.e. 1st and 2nd visit; 2nd and 3rd visit etc.). Continuous variables are presented as means (SD) and categorical variables using number (%). Normal distribution was assessed by the Shapiro–Wilk test prior to any parameter analysis. Patients were divided into tertiles according to the pattern of QRS width progression and differences between groups analysed by ANOVA or, in those with nonparametric distribution, by the Kruskal–Wallis test, respectively. Differences between groups of categorical variables were analysed by Fisher’s exact test. Survival curves were calculated for the prespecified outcome for each tertile and compared by means of the log-rank test.

Markers for QRS width progression were a-priori identified based on statistical significance in the baseline table. Further explorative analysis was omitted to prevent errors by multiple testing. Thresholds for baseline markers that identify patients at increased risk of QRS width progression were derived from an age- and sex-adjusted Cox model by restricted cubic splines (RCS). Thresholds were selected when confidence interval of hazard ratios for QRS progression significantly deviated from 1.0. In addition, each threshold was confirmed in a stepwise adjusted Cox-model adjusted for age and sex (Model 1), IVSd (Model 2) and baseline heart failure therapy (including doses of beta-antagonist, renin–angiotensin inhibition, SGLT2 inhibitor mineralocorticoid receptor antagonist and Ivabradine; Model 3). Where Cox regression was performed, the global test for the proportional hazard assumption by scaled Schönfeld residuals provided a *p* value < 0.05.

In an explorative approach, dynamic changes of baseline table parameters and their association with QRS progression over time were assessed based on tertiles by the Kruskal–Wallis test for difference between groups. In addition, Spearman Rho was calculated to test for trend. All analyses were performed using R (version 4.0.2) with packages ‘survival’, ‘survminer’, ‘rms’, ‘ggplot2’ and ‘gtsummary’, [11]. All statistical tests were 2-sided and p values < 0.05 were considered significant.

## Results

In total 240 datasets were generated from 100 patients with a mean time between consecutive visits of 227 (427) days. Table 1 shows the baseline characteristics divided by tertile of QRS width progression. Patients with QRS width progression (3rd tertile) had a mean increase in QRS duration of 3.57 (10.11) ms/month compared with those classified as regressors (1st tertile in whom QRS change was -1.65 (1.50)ms/month), or stable (2nd tertile in whom QRS change was 0.03 (0.19)ms/month).

**Table 1:** Clinical characteristics of 240 datasets generated from 100 patients stratified into tertiles according to QRS width progression/month

Patient characteristics	Overall	1st Tertile	2nd Tertile	3rd Tertile	*p* value^1^
		< − 0.34 ms/month	− 0.33 to 0.49 ms/month	> 0.50 ms/month	
*N*	240	80	80	80	
Age (years)	58 (13)	58 (12)	58 (13)	59 (14)	0.52
Sex					
Male	191/240 (80%)	63/80 (79%)	66/80 (82%)	62/80 (78%)	0.72
Female	49/240 (20%)	17/80 (21%)	14/80 (18%)	18/80 (22%)	
NYHA class	1.79 (0.63)	1.76 (0.59)	1.77 (0.67)	1.84 (0.63)	0.66
Days between Visits	227 (427)	143 (85)	400 (699)	138 (96)	** < 0.001**
ECG					
QRS progression (ms)	1 (9)	− 7 (6)	1 (5)	8 (8)	** < 0.001**
QRS Progression/month (ms)	0.65 (6.27)	− 1.65 (1.50)	0.03 (0.19)	3.57 (10.11)	** < 0.001**
QRS (ms)	106 (18)	107 (19)	105 (17)	105 (18)	0.92
QRS morphology					
None	171/239 (72%)	57/80 (71%)	58/80 (72%)	56/79 (71%)	0.7
LBB	28/239 (12%)	9/80 (11%)	12/80 (15%)	7/79 (8.9%)	
RBB	13/239 (5.4%)	6/80 (7.5%)	3/80 (3.8%)	4/79 (5.1%)	
Incomplete LBB	25/239 (10%)	8/80 (10%)	6/80 (7.5%)	11/79 (14%)	
Incomplete RBB	1/239 (0.4%)	0/80 (0%)	0/80 (0%)	1/79 (1.3%)	
Undefined	1/239 (0.4%)	0/80 (0%)	1/80 (1.2%)	0/79 (0%)	
PR interval (ms)	171 (31)	174 (31)	168 (26)	173 (35)	0.57
Heartrate (min)	69 (12)	67 (11)	67 (11)	72 (13)	**0.017**
Rhythm					
Sinus rhythm	227/240 (95%)	78/80 (98%)	76/80 (95%)	73/80 (91%)	0.25
Atrial fibrillation	13/240 (5.4%)	2/80 (2.5%)	4/80 (5.0%)	7/80 (8.8%)	
Device					
ICD	69/240 (29%)	21/80 (26%)	25/80 (31%)	23/80 (29%)	0.88
sICD	8/240 (3.3%)	2/80 (2.5%)	4/80 (5.0%)	2/80 (2.5%)	
Medical history					
Diabetes	52/240 (22%)	18/80 (22%)	16/80 (20%)	18/80 (22%)	0.91
Chronic kidney disease	63/240 (26%)	24/80 (30%)	18/80 (22%)	21/80 (26%)	0.56
Coronary artery disease	119/240 (50%)	38/80 (48%)	39/80 (49%)	42/80 (52%)	0.81
COPD	22/240 (9.2%)	8/80 (10%)	6/80 (7.5%)	8/80 (10%)	0.82
Stroke	11/240 (4.6%)	2/80 (2.5%)	7/80 (8.8%)	2/80 (2.5%)	0.12
Hypertension	130/240 (54%)	45/80 (56%)	39/80 (49%)	46/80 (57%)	0.49
Heart failure aetiology					
ICM	113/240 (47%)	34/80 (42%)	38/80 (48%)	41/80 (51%)	0.54
Non-ICM	127/240 (53%)	46/80 (57%)	42/80 (52%)	39/80 (49%)	
Echocardiography					
IVSd (mm)	10.58 (2.14)	10.71 (1.74)	10.22 (2.03)	10.79 (2.56)	0.14
Ejection Fraction	36 (10)	37 (9)	36 (8)	35 (11)	0.34
RVSP (mmHg)	26 (13)	23 (9)	26 (13)	28 (14)	0.13
LVEDd (mm)	58 (8)	57 (6)	59 (8)	58 (9)	0.47
LVESd (mm)	47 (10)	45 (9)	48 (10)	47 (12)	0.22
Laboratory parameters					
Haemoglobine (mmol/L)	13.95 (1.74)	13.96 (1.69)	14.15 (1.64)	13.75 (1.88)	0.48
Sodium (mmol/L)	139.00 (3.12)	138.61 (3.29)	139.09 (2.89)	139.29 (3.18)	0.34
Potassium (mmol/L)	4.55 (0.43)	4.54 (0.47)	4.51 (0.40)	4.61 (0.43)	0.28
NTproBNP (pg/ml)	861 (1,775)	523 (565)	672 (1,087)	1,389 (2,757)	**0.008**
Uric acid (µmol/L)	6.50 (1.96)	6.59 (1.94)	6.51 (2.04)	6.40 (1.92)	0.80
eGFR (ml/min/1.73m^2^)	73 (24)	69 (23)	75 (25)	73 (23)	0.33
Creatinine (mg/dl)	1.16 (0.48)	1.19 (0.42)	1.16 (0.53)	1.13 (0.50)	0.24
HbA1c (%)	6.13 (1.22)	6.32 (1.22)	6.01 (1.38)	6.07 (1.08)	0.47
Medication					
Betablocker	227/238 (95%)	75/79 (95%)	75/79 (95%)	77/80 (96%)	0.86
Dose, Mean (SD)	42 (26)	42 (27)	46 (27)	39 (25)	0.21
Mineralreceptor antagonist	175/238 (74%)	58/79 (73%)	61/79 (77%)	56/80 (70%)	0.59
Dose, Mean (SD)	37 (29)	38 (28)	40 (30)	35 (28)	0.63
Sacubitril/valsartan	128/238 (54%)	45/79 (57%)	39/79 (49%)	44/80 (55%)	0.61
Dose, Mean (SD)	31 (37)	35 (39)	28 (35)	31 (38)	0.48
ACE inhibitor/ AT2 receptor antagonist	102/238 (43%)	33/79 (42%)	37/79 (47%)	32/80 (40%)	0.67
Dose, Mean (SD)	22 (32)	22 (34)	24 (30)	18 (31)	0.37
SGLT-2 inhibitor	45/238 (19%)	13/79 (16%)	17/79 (22%)	15/80 (19%)	0.72
Ivabradine	34/238 (14%)	8/79 (10%)	16/79 (20%)	10/80 (12%)	0.17
Loop-diuretic	167/238 (70%)	53/79 (67%)	52/79 (66%)	62/80 (78%)	0.21
Dose eq furosemid, mean (SD)	15 (28)	14 (29)	12 (17)	19 (35)	0.31
Amiodaron	9/238 (3.8%)	2/79 (2.5%)	2/79 (2.5%)	5/80 (6.2%)	0.52
Aspirin	81/238 (34%)	25/79 (32%)	29/79 (37%)	27/80 (34%)	0.80
Statin	140/238 (59%)	47/79 (59%)	44/79 (56%)	49/80 (61%)	0.77
Anticoagulation	104/238 (44%)	34/79 (43%)	34/79 (43%)	36/80 (45%)	0.96

Continuous data were tested by ANOVA and Kruskal–Wallis test in case of non-normally distributed data. Categorical data were tested by Fisher’s exact test

Bold values indicate a *p*-value of at least < 0.05

^1^Kruskal-Wallis rank sum test; Fisher's exact test; Pearson's Chi-squared test

Assessment of differences in baseline characteristics between the groups revealed that patients with QRS width progression (3rd tertile) had a higher baseline heart rate (*p* = 0.007) and NTproBNP plasma levels (*p* = 0.008). Other features including aetiology, co-morbidities and medical therapy were broadly similar. Of note, the dose of beta-antagonists and Ivabradine between these groups was not different despite the finding of a higher heart rate in the highest tertile.

### Incidence of clinical endpoints in patients with QRS progression

**Fig. 1:**
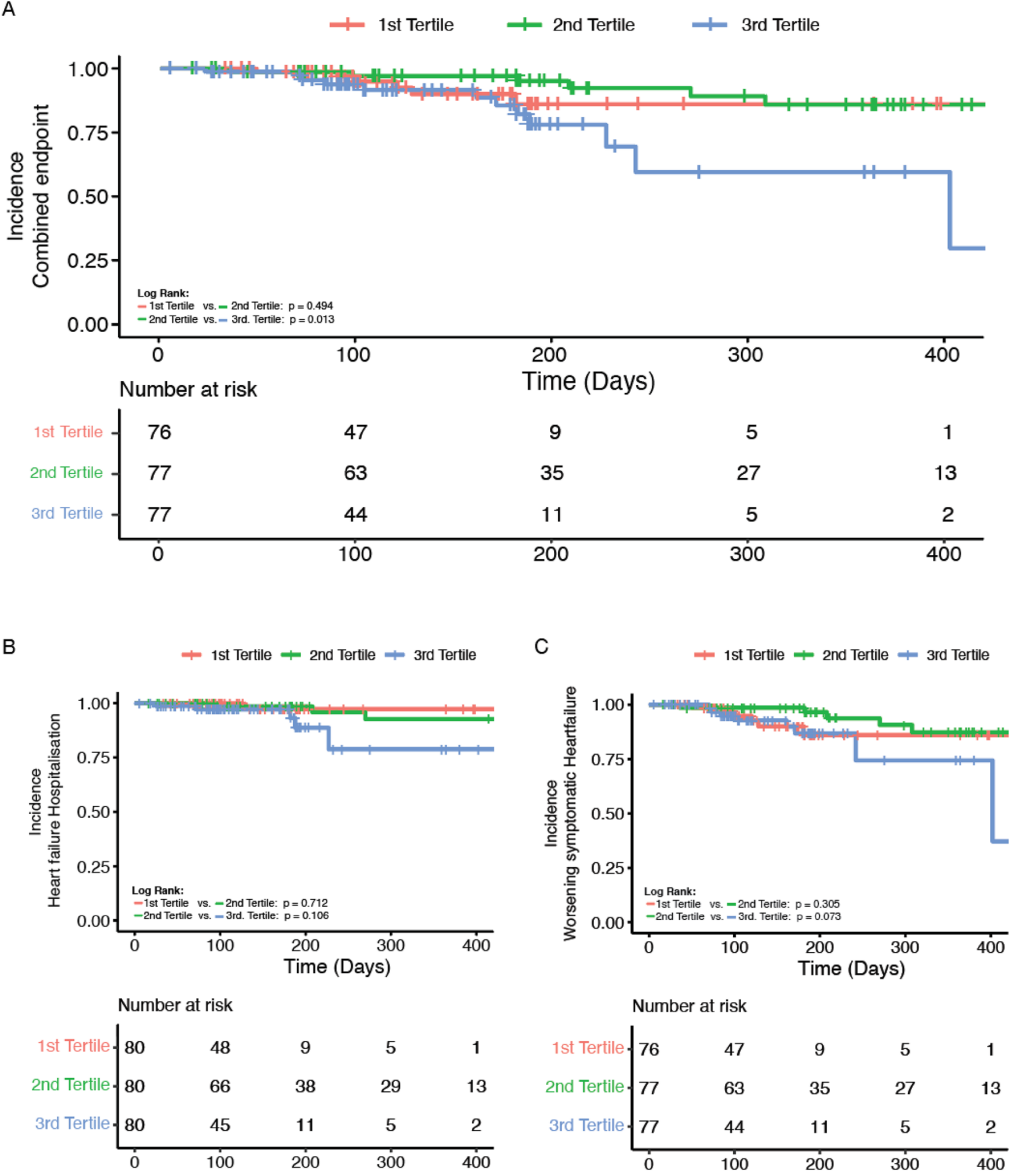
Kaplan–Meier survival plot for 240 datasets. Datasets were stratified into tertiles according to QRS width progression/month

The primary combined outcome (combination of hospitalisation for heart failure or deteriorating symptoms) occurred in 27/240 datasets (1st tertile: 6 [7.9%]; 2nd tertile 9 [12%]; 3rd tertile 12 [16%]) during a mean follow-up between visits of 227 days. Figure 1 shows the Kaplan–Meier curve for freedom from the primary combined outcome. There was a significant difference in event rate between those that develop QRS width progression (3rd tertile) and those that have no overall change in QRS duration (2nd tertile, *p* = 0.013) although the curves for each of the two key components of the primary endpoint, hospitalisation for heart failure or worsening symptomatic heart failure do not reach statistical significance alone (*p* = 0.073 and *p* = 0.106; Fig. 1 B + C).

### Baseline heart rate predicts QRS width progression

**Fig. 2:**
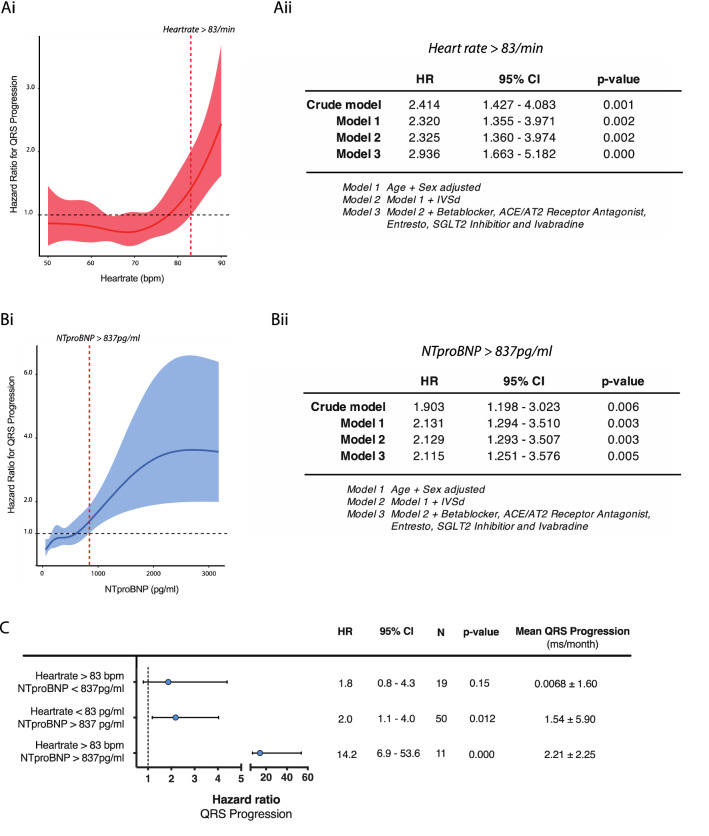
**Ai** Prediction function by restricted cubic splines for the continuous relationship between QRS width progression and heartrate. Restricted cubic splines were used to define cut-offs for patients at risk. Datasets were stratified according to the presence or absence of QRS width progression. **Aii** Cox proportional hazard model for patients with a heart rate > 83/min and QRS width progression stepwise adjusted for age and sex (model 1), IVSd (model 2) and additional guideline directed medical therapy (model 3). **Bi**, **Bii** As in **A** except for NTproBNP. **C** Interaction analysis for heartrate and NTproBNP risk groups by an age- and sex-adjusted Cox proportional hazard model for QRS width progression

The baseline table indicated that heart rate was significantly higher in patients with QRS width progression (3rd tertile) at baseline and Spearman correlation confirmed a linear relationship between QRS progression/month and heartrate (Rho: 0.16; *p* = 0.019). The Cox model for QRS width progression, adjusted for age and sex, identified that patients with a baseline heart rate > 83/min are at increased risk of QRS progression (Fig. 2Bi). Further adjustment for guideline-directed medical therapy and in particular beta-antagonist dose did not change the significant relationship of heart rate with QRS width progression (HR: 2.3 [95% CI 1.39–3.91] *p* = 0.001; Fig. 2Bii). This relationship was not influenced by aetiology of heart failure (ICM vs. nonICM; *p* for interaction = 0.427; S1 Supplementary material). In addition, C-statistic revealed that heart rate was a reliable discriminator of QRS width progression, although the area under the curve (AUC) was modest at 0.581 (95% CI 0.515 – 0.669; *p* = 0.019).

### NTproBNP plasma levels predicts QRS progression

In addition to baseline difference of heart rate, NTproBNP plasma levels were significantly different in patients with QRS width progression and correlation analysis confirmed a significant relationship between QRS width progression/month and NTproBNP plasma levels (Rho 0.17; *p* = 0.009). The sex- and age-adjusted Cox model for QRS progression as a function of NTproBNP plasma levels identified patients levels > 837 pg/ml at increased risk of QRS width progression (Fig. 2Ai). Further adjustment for guideline-directed medical therapy did not affect this relationship (HR 2.44 [95% CI 1.50–3.96], *p* < 0.001; Fig. 2Aii). This relationship was not influenced by aetiology of heart failure (ICM vs. nonICM; *p* for interaction = 0.178; S1 supplementary material). The C-statistic demonstrated a moderate but significant AUC of 0.588 (95% CI 0.511–0.665; *p* = 0.025) for NTproBNP confirming its role as significant discriminator for QRS width progression.

### Heart rate and NTproBNP have a synergistic effect on QRS width progression

Patients with a baseline heartrate > 83 bpm and a baseline NTproBNP > 837 pg/ml were at highest risk of QRS width progression compared to those patients with either marker, thus implying synergism of both variables (HR: 14.2 [95% CI 6.9– 53.6] *p* < 0.0001, *p* for interaction = 0.016; Fig. 2C). Patients within this risk group had the highest mean QRS width progression of 2.2 ms/month (2.2) compared to 1.54 (5.90) ms/month for NTproBNP and 0.0068 (1.60) ms/month for heart rate alone.

### Parameters associated with QRS width progression

QRS width progression was modestly, although statistically significantly associated with an increase in LVEDD (*r* = 0.14; *p* = 0.03, Table 2) and IVSd (*r* = 0.14; *p* = 0.05, Table 2). In addition, patients with QRS width progression were characterised by a significant worsening of renal function (eGFR: Rho: − 0.15; *p* = 0.03 Creatinine: *r* = 0.15; *p* = 0.02) and a decrease in plasma potassium concentrations (*r* = − 0.15; *p* = 0.02).

**Table 2:** Absolute change of clinical characteristics over time stratified into tertiles according to QRS width progression/month. P for trend calculated by Spearman Rho

	Overall	1st Tertile	2nd Tertile	3rd Tertile	*p* value	Rho	*p* value for trend^1^
ECG							
PQ (ms)	1 (15)	1 (13)	1 (15)	1 (18)	0.88	0.00	0.97
Heartrate (/min)	− 1 (9)	− 1 (9)	1 (9)	− 3 (9)	**0.02**	− 0.12	0.06
Echocardiography							
IVSd (mm)	4.00 (1.05)	3.89 (1.06)	3.92 (1.04)	4.18 (1.04)	0.1	0.14	**0.05**
Ejection fraction (%)	0.29 (3.92)	0.19 (4.75)	0.25 (3.61)	0.43 (3.38)	0.92	0.01	0.91
RVSP (mmHg)	1 (13)	4 (14)	1 (11)	− 2 (15)	0.46	− 0.12	0.25
LVEDD (mm)	− 0.4 (4.1)	− 1.4 (3.9)	0.0 (4.1)	0.2 (4.0)	0.06	0.14	**0.03**
LVESD (mm)	− 1 (9)	− 1 (6)	0 (7)	0 (12)	0.37	0.11	0.17
Medication							
Betablocker (%Dose)	0 (14)	1 (14)	0 (16)	0 (12)	0.95	0.02	0.76
Mineralreceptor antagonist (%dose)	0 (15)	3 (12)	− 1 (19)	0 (13)	0.26	− 0.06	0.34
Entresto (%dose)	2 (17)	3 (15)	4 (18)	0 (16)	0.26	− 0.09	0.18
ACE inhibitor/AT2 receptor Antagonist (% Dose)	0 (14)	1 (13)	− 1 (19)	1 (9)	0.64	0.02	0.73
Dose Eq furosemid (mg)	1.9 (15.2)	0.3 (7.7)	2.8 (19.9)	2.5 (15.3)	0.51	0.07	0.28
Laboratory parameters							
Haemoglobine (mmol/L)	30 (18)	29 (18)	30 (17)	32 (19)	0.46	0.07	0.26
Sodium (mmol/L)	0.6 (9.9)	2.3 (16.1)	− 0.1 (2.7)	− 0.3 (4.5)	0.33	− 0.10	0.15
Potassium (mmol/L)	0.03 (0.66)	0.13 (0.70)	0.04 (0.76)	− 0.07 (0.49)	0.07	− 0.15	**0.02**
NTproBNP (pg/ml)	− 44 (1,410)	21 (516)	143 (1,902)	− 297 (1,435)	0.23	− 0.07	0.29
Uric acid (µmol/L)	0.02 (1.75)	0.12 (1.31)	− 0.11 (2.38)	0.06 (1.42)	0.87	0.00	0.97
eGFR (ml/min/1.73m^2^)	− 1 (12)	1 (11)	1 (12)	− 3 (13)	0.08	− 0.15	**0.03**
Creatinine (mg/dl)	0.05 (0.30)	0.00 (0.28)	0.03 (0.20)	0.13 (0.38)	0.08	0.15	**0.02**
HbA1c (%)	0.09 (1.77)	0.41 (2.15)	− 0.53 (1.70)	0.25 (1.32)	0.48	− 0.03	0.79

Bold values indicate a *p*-value of at least < 0.05

^1^Spearman rho

## Discussion

The current study demonstrates that QRS width progression is linked to an increased incidence of the combined clinical endpoint comprising (i) hospitalisation for heart failure in an outpatient clinic setting and (ii) worsening symptomatic heart failure. Furthermore, we demonstrate that QRS width progression is associated with both higher baseline heart rate and higher baseline NTproBNP with the presence of either of these independently increasing the risk of QRS width progression over time. Validation of these variables in prospective trials may help to identify patients that may benefit earlier from a careful assessment of how their indication for device therapy might evolve.

It is well established that a prolonged QRS complex duration > 120 ms is related to worse LV function and clinical outcomes[12–14]. Previous studies have suggested a link between change in QRS duration and major adverse cardiovascular events (MACE) in heart failure, congenital heart disease, atrial fibrillation and unselected male populations[15–19]. The present data support these results and demonstrate, in a real-world setting, that progression of QRS duration is related to a risk of and deteriorating of heart failure. In contrast to previous studies that focused primarily on the prognostic role of QRS width progression, we took a different approach and identified potential predictors that indicate patients at risk of and rate of QRS progression specifically with a view to optimising device choice. In the current study, baseline NTproBNP plasma levels > 837 pg/ml and baseline heart rate > 83/bpm identified patients at increased risk of QRS progression irrespective of medical therapy. Furthermore, we observed a strong interaction between these parameters and presence of both risk factors increased the risk of QRS progression 14-fold with a mean QRS progression of 2.2 ± 2.2 ms/month compared to patients with lower NTproBNP plasma levels and heart rate. Of note, our data are in line with previous observations that linked NTproBNP levels > 1000 pg/ml to progressive LV remodelling[20] and reflect to those of Xiao et al. which demonstrated that in patients with dilated cardiomyopathy QRS width progression is independent of medical therapy[15]. Critically, the rapid progression of remodelling and worse prognosis seen in people with broad QRS, the lack of reverse remodelling in response to optimal therapy in this group in the absence of CRT[3], and the solid evidence of beneficial remodelling apparent following CRT[21] underlines a close link between the QRS duration and LV remodelling.

Despite the link to deteriorating heart failure, also evidenced by the relationship between QRS progression and renal dysfunction[22], the aetiology and drivers of QRS broadening are unclear. Dobutamine stress testing provoked QRS prolongation in patients with pre-existing heart disease but not in healthy subjects and suggests one mechanism could be the failure of the myocardium to adapt to stress[23]. On the other hand, longer-term changes, leading to a gradual extension of the QRS duration, could include progressive fibrosis[24], increasing left ventricular mass[25] and progressive left ventricular diastolic dimensions [26]. In line with these observations, we found a link between QRS progression and progression of left ventricular end diastolic diameter (LVEDD) and interventricular septum diameter (IVSd) as marker of remodelling.

Our study has several strengths and limitations. Firstly, patients were admitted to our specialised outpatient heart-failure clinic. Therefore, our cohort represents patients with a mixture of heart failure aetiologies at different stages and may have suffered bias by patient selection. Secondly, due to the retrospective nature of this study the current analysis may have suffered additional bias and will need prospective validation of our data. Therefore, the current study should be considered as primarily hypothesis generating. Nevertheless, we present real-world data on consecutively selected patients that underline the prognostic potential of changes in QRS duration over time. In addition, our approach to analyse patient datasets instead of individual patients increases power and precision of our models to identify risk factors for QRS progression. Of note our results confirm previous results and underline its validity.

### Conclusions

Progression in QRS width over time is linked to clinical deterioration of symptomatic heart failure. Elevated levels of NTproBNP and a higher baseline heart are associated with QRS width progression independent of guideline directed medical therapy. Future work is needed to confirm these preliminary findings and to clarify whether patients could benefit from modified device treatment strategies.

## Supplementary Information

Below is the link to the electronic supplementary material.

Supplementary file1 (DOCX 1550 KB)

## Data Availability

The data presented in this study are available on request from the corresponding author.
