# Supplementary material for: Clinical implications and risk factors for QRS prolongation over time in heart failure patients
Source: Clin Res Cardiol. 2022 Nov 15;112(2):312–22. doi: 10.1007/s00392-022-02122-y (PMC9898415; doi:10.1007/s00392-022-02122-y)
Supplement: Supplementary file 1 — Supplementary file1 (DOCX 1550 KB) [file 392_2022_2122_MOESM1_ESM.docx]

**Supplementary material:**

Table S3: Patient characteristics at baseline for non-ICM patients; stratified in tertiles by QRS Progression/month

Table S4: Patient characteristics at baseline for ICM patients; stratified in tertiles by QRS Progression/month
